# Cryptic and ubiquitous aplastidic cryptophytes are key freshwater flagellated bacterivores

**DOI:** 10.1038/s41396-022-01326-4

**Published:** 2022-10-07

**Authors:** Karel Šimek, Indranil Mukherjee, Tiberiu Szöke-Nagy, Markus Haber, Michaela M. Salcher, Rohit Ghai

**Affiliations:** 1 Biology Centre CAS, Institute of Hydrobiology, Na Sádkách 7, 370 05 České Budějovice, Czech Republic; 2 University of South Bohemia, Faculty of Science, Branišovská 31, 370 05 České Budějovice, Czech Republic

**Keywords:** Water microbiology, Microbial ecology

## Abstract

Morphology-based microscopic approaches are insufficient for a taxonomic classification of bacterivorous heterotrophic nanoflagellates (HNF) in aquatic environments since their cells do not display reliably distinguishable morphological features. This leads to a considerable lack of ecological insights into this large and taxonomically diverse functional guild. Here, we present a combination of fluorescence in situ hybridization followed by catalyzed reporter deposition (CARD-FISH) and environmental sequence analyses which revealed that morphologically indistinguishable, so far largely cryptic and uncultured aplastidic cryptophytes are ubiquitous and prominent protistan bacterivores in diverse freshwater ecosystems. Using a general probe for Cryptophyceae and its heterotrophic CRY1 lineage, we analyzed different water layers in 24 freshwater lakes spanning a broad range of trophic states, sizes and geographical locations. We show that bacterivorous aplastidic cryptophytes and the CRY1 lineage accounted for ca. 2/3 and ¼ of total HNF, respectively, in both epilimnetic and hypolimnetic samples. These heterotrophic cryptophytes were generally smaller and more abundant than their chloroplast-bearing counterparts. They had high uptake rates of bacteria, hinting at their important roles in channeling carbon flow from prokaryotes to higher trophic levels. The worldwide ubiquity of Cryptophyceae and its CRY1 lineage was supported by 18S rRNA gene sequence analyses across a diverse set of 297 freshwater metagenomes. While cryptophytes have been considered to be mainly plastidic “algae”, we show that it is the aplastidic counterparts that contribute considerably to bacterial mortality rates. Additionally, our results suggest an undiscovered diversity hidden amongst these abundant and morphologically diverse aplastidic cryptophytes.

## Introduction

Aquatic microbial food webs and carbon flow processes have been in the scientific focus since the recognition of the microbial loop [1] that recognized main trophic interactions based on bacterial utilization of dissolved organic carbon, thereby producing particulate biomass that is in turn consumed by small protists [2–4]. There has been a considerable expansion of our understanding of metabolic traits and recurrent temporal cycles of major prokaryotic groups in a broad variety of freshwater environments [5–9]. However, the community composition of unicellular eukaryotes, particularly those that are small, planktonic and largely aplastidic, i.e., heterotrophic nanoflagellates (HNF), is much less understood. Most freshwater bacterivorous HNF are barely distinguishable microscopically, they are usually small (2–8 µm in size), oval- to drop-shaped cells, with a single nucleus and one or two distinct flagella. This “morphological homogeneity” obscures the diversity of such largely uncultured protists and limits reliable species detection using distinctive morphological features [10–13].

In freshwater plankton, several easily cultivable bacterivorous HNF, such as chrysophytes (*Spumella*-like morphotypes), bodonids and Cercozoa, have been assumed to represent dominant bacterivorous HNF [11, 12, 14, 15]. Similar assumptions have also been made in marine systems, especially with regard to cultivated bacterivorous flagellates such as the chrysophyte *Paraphysomonas* spp. that have been frequently used in laboratory experiments [16, 17]. However, it is becoming increasingly evident that the vast majority of HNF in both marine and freshwaters remain uncultured and that the easily cultivable members of this functional guild may not represent abundant pelagic flagellated bacterivores [18–22].

The increased use of amplicon-sequencing accelerated our understanding of the prevalence of dominant groups, but data resolution remains limited owing to discrepancies in 18S rRNA reference databases. For instance, most of aplastidic cryptophytes are uncultured although there is an increasing evidence that they belong to prominent freshwater HNF, especially the so-called CRY1 lineage [18, 22]. They have not been recognized as bacterivores for a long time, with the exception of relatively easily cultivable species of the genus *Goniomonas* [23] and *Chilomonas* [24]. One reason could be that while 18S rRNA sequence data allow placing organisms in a phylogenetic context, however, in particular groups such as Cryptophyceae they do not provide sufficient information on the presence or absence of chloroplasts in cells and thus bring rather limited information on their lifestyle. Most of the so far known cryptophyte-related 18S rRNA gene sequences have been attributed to chloroplast-bearing autotrophic or mixotrophic members of the cryptophyte group [25–29]. Moreover, some cryptophyte groups as the aplastidic CRY1 lineage are underrepresented in amplicon sequencing data when compared to microscopically quantified cryptophyte cells targeted by specific FISH probe in the same samples [30].

To unveil taxonomic affiliation, abundance, and ecological traits of the major taxa in situ, a renewed research strategy, using new isolation and cultivation methods and single-cell molecular approaches such as CARD-FISH [21, 31] in combination with prey tracer techniques, have been proposed [18, 19, 22, 32]. A surprising outcome of applying these method combinations in situ was the discovery that tiny aplastidic Cryptophyceae and its monophyletic CRY1 lineage [33] were prominent flagellate bacterivores in e.g., a meso-eutrophic reservoir [18, 34] and shallow hypertrophic freshwater lakes [22, 34]. However, to draw general conclusions, these discoveries need to be confirmed in a broad variety of freshwater habitats and supported by metagenomic analyses to address the following intriguing questions: Why were such abundant primarily bacterivorous protistan groups not recognized earlier and whether these HNF groups are ubiquitous in freshwater habitats of different geography and trophic states?

In this study, we investigated distribution patterns and the ecological role of phagotrophic aplastidic cryptophytes and the CRY1 lineage in 24 freshwater habitats using the CARD-FISH approach, in selected cases accompanied by fluorescently labeled bacteria as prey tracers. These single-cell detection techniques were complemented by a detailed meta-analysis of cryptophyte-related 18S rRNA gene sequences from a large number of metagenomes from freshwater but also marine habitats spanning a broad geographical (five continents), size (small and shallow lakes to large and deep lakes), and trophic range (ultra-oligotrophic to hypertrophic). We hypothesized that aplastidic cryptophytes are ubiquitous and abundant key freshwater bacterivores.

## Materials and methods

### Study sites and field sampling of freshwater habitats

To cover a broad diversity of freshwater habitat types, the proportions of bacterivorous aplastidic cryptophytes, its CRY1 lineage and of total HNF abundances were studied in 24 freshwater lakes of different trophic state (based on total phosphorus and Chl-*a* concentrations [35–37]), size, altitude, maximum depth, and geological origin classified as: alpine lakes (*n* = 2), deep prealpine lakes (*n* = 10), a caldera lake (*n* = 1), a deep tectonic lake (*n* = 1), shallow hypertrophic lakes (*n* = 4), mine pit lakes (*n* = 3), and dam reservoirs (*n* = 3) (for details of lake classification and corresponding background data see Table 1). Nineteen habitats were located in Europe and five large lakes in Japan (Table 1 and Supplementary Table S1). Water samples from 24 epilimnetic and for 14 lakes also hypolimnetic layers were taken at varying seasons, from single summer samples to full seasonal profiles summing up to a total of 112 samples (for details of sampling see Supplementary Table S1). Epilimnetic water layers of eight habitats were studied seasonally, covering different aspects of the seasonal plankton succession: the oligo-mesotrophic lake Biwa was sampled monthly (see Supplementary Fig. S1), three oligo-meso to eutrophic dam reservoirs (Klíčava, Římov and Žlutice) in Czech Republic were sampled in April, June, August and October 2019, and four shallow hypertrophic lakes were sampled monthly (data taken over from [22]).

**Table 1:** Parameters characterizing geographic locations with GPS coordinates, altitude, mean and maximum depths and area of the 24 freshwater habitats studied by using CARD-FISH probes targeting all cryptophytes (CryptoB, [27]) and the CRY1 lineage (Cry1-652, [18]), presented on the basis of their increasing Chl-*a* concentrations and total phosphorus in epilimnetic water layers from ultra-oligotrophic to hypertrophic ones.

Lake/habitat	Country	Coordinates	Trophic state	Altitude (ASL)	Max depth (*Z*_max_)	Mean depth (*Z*_m_)	Area (km^2^)	Chl-*a* (µg l^−1^)	Total P (µg l^−1^)
Gossenköllesee (AL)	Austria	47°13'46.0”N 11°00'50.0”E	Ultra-oligotrophic	2417	9	4.7	0.015	0.39	1.2^c^
Attersee (DPL)^a^	Austria	47°52'00.0”N 13°32'00.0”E	Ultra-oligotrophic	469	171	25	46.2	0.60	1.5^c^
Motosu (DPL)	Japan	35°27'50.3”N 138°35'01.9”E	Ultra-oligotrophic	897	122	68	4.83	0.95	2–5^d^
Medard (MPL)^a^	Czech Republic	50°10'45.9”N 12°35'15.4”E	Oligotrophic	400	50	24	4.90	1.24	6.8
Traunsee (DPL)^a^	Austria	47°52'00.0”N 13°48'00.0”E	Oligotrophic	423	191	95	24.4	1.51	2.6^c^
Lago Maggiore (DPL)^a^	Italy	45°58'00.0”N 8°39'00.0”E	Oligo-mesotrophic	194	370	177	212.5	1.59	2.9^c^
Thun (DPL)^a^	Switzerland	46°41'00.0”N 7°43'00.0”E	Oligotrophic	558	217	136	47.7	1.68	1.9^c^
Sai (DPL)	Japan	35°29'60.0”N 138°41'11.7”E	Oligotrophic	900	71.7	34.8	2.10	1.94	3–8^d^
Most (MPL)^a^	Czech Republic	50°32'13.0”N 13°38'40.0”E	Oligo-mesotrophic	199	75	23	3.09	2.08	7.8
Biwa (DTL)	Japan	35°21'38.2”N 136°10'03.8”E	Oligo-mesotrophic	80	104	45.5	674	2.86	5–9^d^
Chūzenji (AL)	Japan	36°44'12.2”N 139°28'56.8”E	Oligo-mesotrophic	1269	163	95	11.5	2.86	3–9^d^
Ikeda (CL)	Japan	31°14'11.8”N 130°33'48.6”E	Oligo-mesotrophic	88	233	125	11.0	3.11	3–6^d^
Klíčava (DR)^a^^,^^b^	Czech Republic	50°03'58.0”N 13°55'56.0”E	Oligo-mesotrophic	269	35	13	0.53	4.53	13.3
Constance (DPL)^a^	Germany	47°38'00.0”N 9°22'00.0”E	Mesotrophic	395	252	100	536	4.60	2.9^c^
Lugano (DPL)^a^	Switzerland	45°59'00.0”N 8°58'00.0”E	Mesotrophic	271	288	171	27.5	4.60	4.4^c^
Milada (MPL)^a^	Czech Republic	50°39'13.0”N 13°56'40.0”E	Mesotrophic	146	25	14	2.52	5.80	20.4
Mondsee (DPL)^a^	Austria	47°49'00.0”N 13°22'00.0”E	Mesotrophic	483	68	34	14.2	7.21	4.8^c^
Zurich (DPL)^a^	Switzerland	47°18'00.0”N 8°34'00.0”E	Mesotrophic	406	137	49	67.3	7.81	5.4^c^
Římov (DR)^a^^,^^b^	Czech Republic	48°50'54.7”N 14°29'26.6”E	Meso-eutrophic	470	45	16	2.10	9.74	20.9
Žlutice (DR)^a^^,^^b^	Czech Republic	50°05'18.0”N 13°07'40.0”E	Eutrophic	511	21	7.7	1.38	11.8	21.2
Dehtář (SHL)^b^	Czech Republic	49°00'28.8”N 14°17'58.0”E	Hypertrophic	406	5.5	2.2	2.28	97.3	230
Kvítkovický (SHL)^b^	Czech Republic	48°57'50.1”N 14°20'11.2”E	Hypertrophic	424	3	1.1	0.24	174	250
Klec (SHL)^b^	Czech Republic	49°05'26.9”N 14°45'59.4”E	Hypertrophic	420	2	0.9	0.64	205	260
Rod (SHL)^b^	Czech Republic	49°07'19.4”N 14°44'42.7”E	Hypertrophic	430	2.2	0.6	0.22	224	360

Classification of hypertrophic shallow lakes follows the criteria suggested in Scheffer [36].

Lake classification: *AL* alpine lake, *DPL* deep prealpine lake, *CL* caldera lake, *DTL* deep tectonic lake, *SHL* shallow hypertrophic lake, *MPL* mine pit lake, *DR* dam reservoir.

^a^Habitats at which also hypolimnetic samples were analyzed (for details see Supplementary Table S1).

^b^Habitats at which bacterivory rates of total HNF and the probe-defined cryptophyte groups were studied using FLB tracers.

^c^Total phosphorus (the last column) was detected as total dissolved phosphorus in the 0.2-µm filtered fraction or in unfiltered water samples (no index) at the sampling date.

^d^In case neither was available, the annual range of total phosphorus values is listed for the epilimnion of the Japanese lakes recorded during 2015 [37].

Unfiltered subsamples (80–120 ml) from the lakes were fixed with formaldehyde (2% final concentration) for prokaryote and HNF quantification with the following exceptions: To effectively retain food vacuole contents of small bacterivorous HNF in samples for CARD-FISH in combination with FLB tracer uptake studies conducted in seven lakes (for details see Table 1), samples were preserved with the Lugol-formol-thiosulphate decolorization technique [38, 39]. Note that most freshwater bacterivorous nanoflagellates (by definition protists 2–20 µm in size) are microscopically barely distinguishable, having generally a small cell size of only 2–8 µm. The focus of our study was on aplastidic bacterivorous cryptophytes that overlap in size and feeding modes with the majority of small freshwater bacterivorous HNF [11, 12, 14, 15]. However, heterotrophic dinoflagellates were not involved as the target protistan group in our study, since they are much larger (15–30 µm), microscopically relatively well-distinguishable specific predators of algae and of other protists (e.g., [11, 31]) and do not feed on bacteria. The samples for counting of HNF and probe-targeted cryptophyte groups in CARD-FISH preparations were filtered on 1-µm pore-size filters (Osmonics, Livermore, CA, USA) within 24 h and stored at −20 °C until processed as detailed below.

Oxygen concentration, pH, conductivity, chlorophyll-*a* (Chl-*a*), and temperature were measured by a multiparameter probe YSI EXO2 (Yellow Springs Instruments, USA) for most lakes (except for the reservoirs and shallow hypertrophic lakes listed in Table 1). Total phosphorus, physical background data and Chl-*a* concentrations in the dam reservoirs and hypertrophic shallow lakes were analyzed according to published protocols [9, 40, 41].

### Enumeration of prokaryotes and protists

Samples from dam reservoirs, shallow hypertrophic lakes and all lakes in Japan (see Table 1) were enumerated by microscopy. Duplicate samples (5–20 ml) fixed with formaldehyde were used to enumerate bacteria (0.4–2 ml subsamples) and HNF (4–15 ml subsamples) on 0.2-μm and 1-μm pore-size filters, respectively [41]. The samples were stained with DAPI (4’,6-diamidino-2-phenylindole, final concentration, 0.1 µg ml^−1^) and microbes were counted via epifluorescence microscopy (Olympus BX53; Optical, Tokyo, Japan). Bacterial counts in all other samples were obtained with a CytoFLEX flow cytometer (Beckman Coulter; Brea, CA, USA) equipped with a blue laser (bandpass filters 525/40 and 690/50) after staining with SYBRGreen I (0.5 × standardized concentration; Lonza, Rockland, ME, USA). Prokaryotic cells were detected in plots of 90° side scatter versus fluorescence intensity and manual gates were applied to distinguish prokaryotes from background noise and larger particles as outlined in Gasol and del Giorgio [42].

### Estimates of flagellate grazing rates on bacteria

In three dam reservoirs and four shallow hypertrophic lakes (see Table 1) the bacterivory rates of all HNF and aplastidic cryptophytes (see below) were estimated using fluorescently labeled bacteria (FLB) prepared according to the protocol of Sherr et al. [43], using a mixture of freshwater bacterial isolates well mimicking typical cell sizes of bacterioplankton (for details see [22]). FLB tracers were added to water samples (300 ml) to constitute 6–16% of total bacteria (the amount of FLB added depended on water temperature) and samples were incubated at in situ temperatures for 30 min. The incubations were terminated with the Lugol-formol-thiosulphate decolorization technique [38]. Subsamples of 2–15 ml were stained with DAPI, passed through 1-µm filters (Osmonics) and >200 HNF individuals were inspected via epifluorescence microscopy to count ingested FLB in HNF at ×1000 magnification [22].

### CARD-FISH detection of aplastidic cryptophytes and their bacterivory rates

**Fig. 1:**
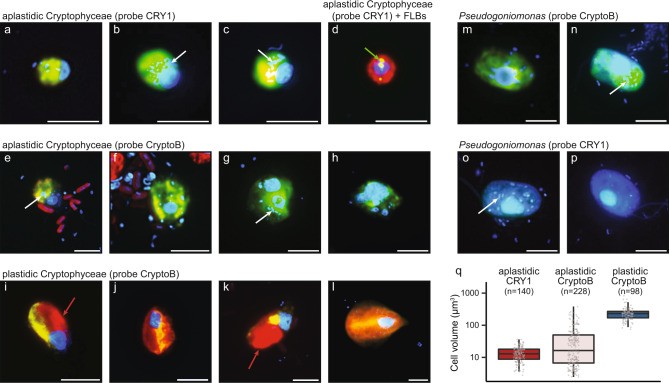
Morphological diversity of cryptophytes and comparative cell volume estimations. Microphotographs showing typical flagellate morphologies and sizes of the cells targeted by two eukaryotic FISH probes, Cry1-652 (**a**–**d**) and CryptoB (**e**–**p**), with ingested DAPI-stained bacterial prey (**a**–**c**, **e**–**h**, **m**–**p**) or FLB (**d**) in food vacuoles. Examples are shown from the studied lakes (**a**–**l**) or a cultured *Pseudogoniomonas* strain (**m**–**p**) isolated from Římov reservoir. Shown are overlay Z-stack images of flagellates targeted by the FISH probes (fluorescein-stained flagellates (yellow) in **a**–**c**, **e**–**p**, or an Alexa546-stained flagellate ([red] in **d**) and DAPI-stained protistan nuclei ([blue] in **a**–**p**), DTAF-labeled ingested FLB ([yellow] in **d**) and chloroplast-bearing autotrophic cells of *Rhodomonas* and *Cryptomonas* spp. with bright orange-red chloroplasts in cells (**i**–**l**). A cultured representative of the aplastidic non-CRY1 cryptophyte *Pseudogoniomonas* sp. (**m**–**p**), targeted by probe CryptoB (**m**, **n**) with a “negative control” of no hybridization signal with probe Cry1-652 (**o**, **p**). White arrows highlight examples of typical positions of ingested bacteria, a green arrow of ingested FLBs (**d**) in the grazer food vacuoles and red arrows positions of chloroplasts (**I**, **k**) in chloroplast-bearing cryptophyte cells. The Z-stack images of protistan cells were acquired with the protocol detailed in Šimek et al. [34, 41]. The scale bar shows a length of 5 μm. Boxplots presenting median and 25th and 75th percentiles of cell volume distributions (**q**) of aplastidic CRY1, aplastidic flagellates targeted by probe CryptoB, and plastidic flagellates targeted by probe CryptoB. Values are based on epilimnetic samples of four lakes where they co-occurred (for details see Supplementary Table S2).

We used two oligonucleotide probes targeting all cryptophytes (CryptoB, [27]) and the CRY1 lineage (Cry1-652, [18]) purchased from Biomers.net (Ulm, Germany, PAGE-type purification). Details on quality and specificity of the FISH-probes are given in a recent review [31]. CARD-FISH was performed at 35 °C for 2–3 h following the protocol described elsewhere [31, 44] with fluorescein labeled tyramides. CARD-FISH preparations were analyzed by epifluorescence microscopy at ×1000 magnification. Cell volumes were calculated based on measurements of width and length of hybridized cells (see Fig. 1), using an image analysis system (NIS-Elements 5.1, Laboratory Imaging, Prague, Czech Republic), as detailed before [34].

Samples from three dam reservoirs and four shallow hypertrophic lakes were also incubated with FLB (see Table 1) to detect uptake rates of bacteria by probe-targeted HNF (the text above, for details see [22]). After incubation with FLB tracers the samples were fixed, concentrated on white 1-µm pore-size filters (47 mm diameter, Osmonics) and stored at −20 °C until further processing by CARD-FISH using Alexa546-labeled tyramides (0.02 mg ml^−1^ [31]). To examine lineage-specific uptake rates of CryptoB and CRY1, their cells (>100 per sample, see examples in Fig. 1a–h) and more than 200 randomly selected DAPI-stained HNF cells (see above) were inspected for FLB uptake using a combination of optical filter sets for excitation of Alexa546-stained flagellates (FISH-positive, red color), DTAF-labeled FLB (yellow) and DAPI-stained nuclei and ingested natural bacteria (blue). To estimate total HNF grazing or contributions of cryptophyte taxa-specific grazing rates, we multiplied average bacterial uptake rates of all HNF, CRY1- and CryptoB-positive cells, respectively, by their in situ abundances. Autotrophic, chloroplast-bearing cryptophytes, which also hybridized with the probe CryptoB were easily discriminated from the heterotrophic ones by their significantly large cells sizes, presence of large chloroplasts (Fig. 1i–l) and, moreover, they did not ingest FLB tracers. These chloroplast-bearing cryptophyte cells were not included into total counts of aplastidic cryptophytes. However, in four selected lakes with simultaneous occurrence of both plastidic and aplastidic cryptophytes they were counted and sized separately (Supplementary Table S2). Images of chloroplast-bearing and aplastidic cryptophyte cells with ingested prey (see Fig. 1) were obtained with a motorized fluorescence microscope Nikon Eclipse 90i (Nikon, Tokyo, Japan) equipped with a monochromatic digital camera Andor Clara (Andor Technology Ltd., Belfast, UK) and the software NIS-Elements 5.11 (Laboratory Imaging) as detailed before [41].

### Statistical analyses

Using Chl-*a* as a proxy of the trophic state of 24 habitats studied (supported by total phosphorus concentrations, see Table 1), the Spearman’s rank correlation analysis was conducted to explain the variability in abundances of bacteria, HNF, probe-defined groups of cryptophytes CryptoB and its CRY1 lineage and of their relative proportions in epilimnetic and hypolimnetic samples (for details see Supplementary Tables S3 and S4, respectively).

### Cultivation of *Pseudogoniomonas*

Water collected from 0.5 m depth from Římov Reservoir (Czech Republic) on April 6th, 2021, was filtered through 10-µm membrane and 1 ml was added to each of five tubes containing 8 ml of sterilized 0.2-µm filtered water and 1 ml of Actinobacteria culture (“*Candidatus* Planktophila versatilis” strain MsE-18, concentrations ca. 2 × 10^7^ cells ml^−1^) as prey. After mixing, 1 ml from each tube was used as inoculum for a second tube (1:100 dilution of the original inoculum). Tubes were incubated in the dark at 16 °C for 2 weeks, flagellate growth was assessed by microscopy using DAPI staining (see above) and cultures were maintained in lake water medium by replacing 1 ml of culture with 1 ml of Actinobacteria culture every 6 to 11 days. The *Pseudogoniomonas* culture was identified by Sanger sequencing of its 18S rRNA gene amplified with primers EukA and EukB [45]. The partial 18S rRNA gene sequence has been deposited at Genbank under accession number ON067811. CARD-FISH was conducted on the *Pseudogoniomonas* culture using probes CryptoB and Cry1-652 (see Fig. 1m–p) as described above.

### Assembly of 18S gene rRNA sequences in metagenomic/metatranscriptomic datasets and phylogenetic tree processing

We used publicly available freshwater metagenomes from a wide variety of samples from Europe, Asia, Africa, and the Americas (*n* = 297, Supplementary Table S5). Additionally, we also used 130 marine metagenomic assemblies from GEOTRACES [46] and 231 metagenomes from TARA Oceans Expeditions [47]. Metagenomic sequences were processed with BBMap tools available from https://github.com/BioInfoTools/BBMap/. Briefly, bbduk.sh script was used to remove poor quality reads (qtrim = rl trimq = 18), and phiX, p-Fosil2 control reads and illumina adapters were removed (*k* = 21 ref = adapterfile ordered cardinality). Cleaned reads were assembled de novo with MEGAHIT v1.2.9 [48] using default parameters and k-mers: 29, 49, 69, 89, 109, 119, 129, 149. All sequences in this work are named or retain existing names that allow tracing them to their original datasets using Supplementary Table S5.

Assembled contigs were scanned using ssu-align (http://eddylab.org/software/ssu-align/) to identify eukaryotic rRNA sequences, which were classified with the SILVA database (v138, [49]) using mmseqs2 [50]. Long sequences that were classified as Cryptophytes, Haptophytes, Katablepharids were collected from the SILVA database and aligned with those recovered from assembled contigs using mafft-linsi [51]. The final phylogenetic tree was created using iqtree2 [52] with ultrafast bootstraps [53] (-bb 1000, -alrt 1000) using automatic model selection by ModelFinder (selected model: TIM3e + I + G4) [54]. Multiple, redundant sequences were removed to retain only the longest and representative sequences from the CRY1 clade and other branches of the tree. All sequences (*n* = 287) used in the tree, their alignment and the final phylogenetic tree are available for download in Figshare (https://figshare.com/s/8540087c6bf5464ff17f).

### Abundance estimations in metagenomes

A subsample of 20 million reads was taken from several freshwater (see Supplementary Table S5) and marine datasets (from Tara Oceans metagenomes) and scanned for eukaryotic rRNA sequences using ssu-align (http://eddylab.org/software/ssu-align/). Eukaryotic rRNA sequences were compared to the SILVA database v138 [49] to identify Cryptophyte sequences using mmseqs2 nucleotide-nucleotide comparisons [50]. For selected freshwater datasets (Supplementary Table S6, datasets marked with asterisk [*]) the entire data set was scanned without any subsampling. Sequences giving best hits to Ciliophora, Diatomea, Metazoa, Dinoflagellata, Nucletmycea, Chytrid, Embryophyta, Charophyta, Ulvophyceae, and Rhodophyceae were excluded from the counts of total eukaryotes in order to retain mainly sequences originating from HNF. Another reason for excluding Ciliophora and Dinoflagellata was that they are known to have hundreds to thousands of rRNA operons [55, 56] that would skew any abundance estimations from 18S rRNA sequence data. Moreover, they also cannot be considered as belonging to the functional guild of nano-sized bacterivorous flagellates. Counts were summarized for sequences matching CRY1 cryptophytes, non-CRY1 cryptophytes and others (see Supplementary Fig. S2 and Supplementary Tables S6 and S7).

## Results

### Ubiquitous occurrence and bacterivory rates of freshwater aplastidic cryptophytes

The CARD-FISH approach allowed visualization of the size and morphology of aplastidic and plastidic Cryptophyceae in different lake types (Fig. 1a–l, and see Table 1 for the lake classification). There is a large diversity in sizes, cell and nucleus morphology of aplastidic cryptophytes targeted by the general probe CryptoB (Fig. 1e–h, q), while Cryptophyceae affiliated to the CRY1 lineage (probe Cry1-652, Fig. 1a–d, q) were small, mostly round-shaped cells (2.5–4.5 µm in size) with relatively uniform morphology of cells and nuclei. All aplastidic phylotypes including the isolated *Pseudogoniomonas* strain were bacterivores with ingested bacteria or FLB (Fig. 1a–h, m–p) in food vacuoles, compared to considerably larger chloroplast-bearing cryptophytes with clearly visible chloroplasts and no apparent bacterial uptake (Fig. 1i–l).

**Fig. 2:**
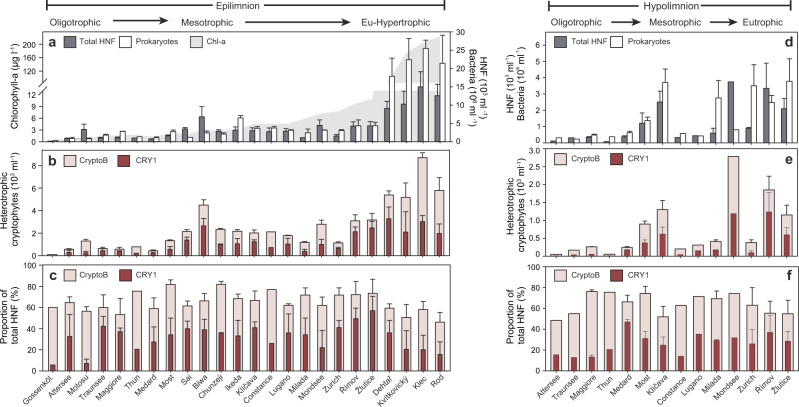
Data from 24 epilimnetic and 14 hypolimnetic freshwater habitats. **a** Chl-*a* concentrations in epilimnion used to arrange the sampling sites by increasing trophic level (left to right, **a**–**f**). Both epi- and hypolimnion water layers: total bacterial and HNF abundance (**a**, **d**), abundance of total aplastidic cryptophytes using general (CryptoB) and CRY1 lineage-specific (Cry1-652) probes (**b**, **e**) and relative proportions of these cryptophyte groups to total HNF (**c**, **f**). Error bars represent the range of values (*n* = 2) or SDs (*n* ≥ 3); for details of the environmental background data and origin of samples see Table 1 and Supplementary Table S1.

**Fig. 3:**
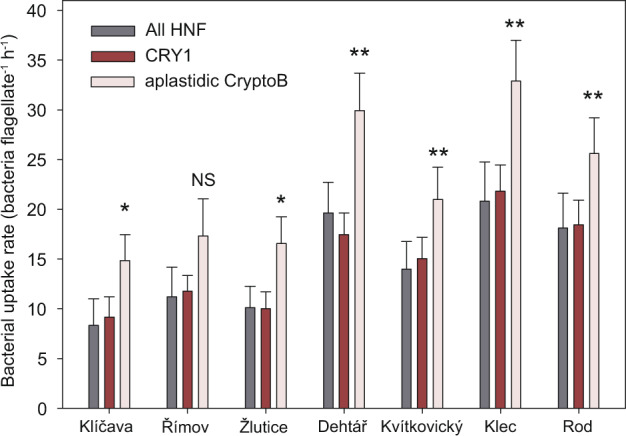
Average cell-specific uptake rates of all HNF and CRY1- and CryptoB-targeted flagellates in samples from three dam reservoirs (Klíčava, Římov and Žlutice) sampled in April, June, August and October 2019, and four shallow hypertrophic lakes sampled monthly (data from [22], see Supplementary Table S1). In all habitats but the Římov Reservoir, CryptoB-targeted flagellates showed significantly higher bacterivory rates (two-tailed paired *t*-test; significance indicated by stars above the columns, *p > 0.05, **p > 0.01) than the average rates detected for all HNF or the CRY1 lineage, while differences in average uptake rates between the latter flagellate groups were insignificant. Error bars show the standard error of the mean; NS not significant.

The largest contribution to our data set investigated by both FISH and metagenomic analyses (Figs. 2 and 3) originates from both epi- and hypolimnion of ten prealpine deep lakes, followed by shallow hypertrophic lakes and mine pit lakes, with the latter two categories being studied also seasonally. Hypolimnetic samples of the oligo-mesotrophic deep lakes were generally taken from fully oxygenated depths. Smaller contributions can be attributed to alpine lakes, a caldera lake and the seasonally studied large tectonic lake Biwa (for details see Table 1, Supplementary Table S1 and Fig. S1). CARD-FISH confirmed the ubiquity of aplastidic cryptophytes (probe CryptoB) and of the CRY1 lineage (probe Cry1-652) at different seasonal phases in both epi- and hypolimnetic water layers of the 24 freshwater habitats spanning extreme oligotrophy to extreme hypertrophy (Fig. 2, Table 1 and Supplementary Table S1). Aplastidic cryptophytes accounted on average (±SD) for 65 ± 9.4% and 64.2 ± 9.6% of total HNF in epi- and hypolimnetic samples, respectively, with maxima of up to 81.9% of all HNF. CRY1 accounted for up to 57% of total HNF (average 31.4 ± 12.4% and 26.2 ± 10.2%, in epi- and hypolimnion, respectively). There was no significant difference between their relative proportions in these two water layers (Welch’s *t*-test, *p* = 0.80 and 0.17 respectively). While the relative proportions of CRY1 oscillated considerably more (minimum 5.7% in Gossenköllesee, maximum 57% in Žlutice reservoir) than that of CryptoB, particularly in the epilimnetic water samples (Fig. 2c), the relative proportions of CRY1 to total aplastidic cryptophytes did not differ significantly between the epi- and hypolimnion of all the lakes (on average 47.9% and 41.9%, *p* = 0.30). Due to relatively stable proportions of aplastidic cryptophytes in all habitats (Fig. 2c, f), their total abundances mainly reflected the total numbers of HNF in different lakes and were highest in four hypertrophic shallow lakes (5.18–8.81 × 10^3^ cells ml^−1^) and the epilimnion of Lake Biwa (4.50 × 10^3^ cells ml^−1^, Fig. 2b). Only moderate seasonal variations of relative proportions of aplastidic cryptophytes and CRY1 were recorded in the Lake Biwa (Supplementary Fig. S1), pointing at an all-year dominance of aplastidic cryptophytes.

Using Chl-*a* as a proxy of the habitat trophy, Spearman’s rank correlation analysis explained 93.3%, 83.5%, 70.1% and 47.5% of the variability in abundances of bacteria, HNF, CryptoB, and CRY1 in epilimnetic samples, respectively (*R*^2^ values of the regression, Supplementary Table S3). Thus, the absolute numbers of both bacterivorous cryptophyte groups (Fig. 2b, e) showed a significantly increasing trend along the Chl-*a* gradient toward hypertrophic lakes. However, relative proportions of CryptoB in the epilimnion were significantly negatively correlated with Chl-*a* concentrations (*R* = −0.569, *p* = 0.004), bacterial (*R* = −0.498, *p* = 0.013) and HNF abundances (*R* = −0.469, *p* = 0.021) and none of these parameters had significant effects on the relative proportions of CRY1 in the epilimnion (Supplementary Table S3).

The absolute abundances of CryptoB and CRY1 tightly correlated in both epi and hypolimnion (*R*^2^ = 0.831 and 0.929, respectively; Supplementary Tables S3 and S4). However, the relative proportions of CryptoB and CRY1 were significantly correlated only in epilimnion (*R*^2^ = 0.178, Supplementary Table S3). This reflects the fact that CryptoB proportions varied only by a factor of 1.7 in epilimnion, while CRY1 proportions oscillated considerably more, i.e., by a factor of 10.1 (Fig. 2c). Hypolimnetic samples displayed less variability in CRY1 proportions (factor of 3.7; Fig. 2e, f) and thus the changes in proportions of CRY1 of CryptoB explained 42.6% of the variability in the absolute numbers of CRY1 (Supplementary Table S4).

The presence of DAPI-stained bacteria in food vacuoles of flagellates (Fig. 1a–c, e–h) and the tracer FLB technique (see example in Fig. 1d) confirmed flagellate bacterivory. Moreover, the uptake of FLB tracers allowed a quantification of cell-specific bacterivory rates of all HNF, CryptoB- and CRY1-targeted HNF (see Fig. 3) in the epilimnion of 3 dam reservoirs (four samples per reservoir, Supplementary Table S1) and in the 4 monthly sampled hypertrophic lakes (see Table 1, data from [22]). In almost all these habitats, CryptoB-targeted flagellates showed significantly (paired *t*-test, *p* < 0.05) higher bacterivory rates (~15–33 bacteria flagellate^−1^ h^−1^) than all HNF or the CRY1 lineage (~8–20 bacteria flagellate^−1^ h^−1^, Fig. 3).

We additionally analyzed cell volumes, total biovolumes and numerical and biovolume ratios of aplastidic to plastidic (e.g., *Rhodomonas* and *Cryptomonas* spp.) cryptophytes targeted by probe CryptoB in the epilimnion of four oligo-mesotrophic lakes with simultaneous occurrence of both types (Supplementary Table S2). Aplastidic cryptophytes, easily distinguishable from plastidic ones, based on size (Fig. 1q) and the absence of chloroplasts (compare Fig. 1a–h with 1i–l), were more abundant by a factor of 2.5–41 than their chloroplast-bearing counterparts. However, plastidic cryptophytes had significantly (Welch’s *t*-test, *p* < 0.001) larger mean cell volumes (factor of 4.6–13.8) than aplastidic ones. Therefore, biovolume ratios of aplastidic:plastidic cryptophytes were lower and plastidic cryptophytes accounted for slightly more biovolume than their aplastidic counterparts in two lakes (1.8 and 3-fold for lakes Milada and Medard, respectively, Supplementary Table S2), while the biovolume of aplastidic ones was clearly higher in the other two lakes (9 and 7.2-fold for lakes Zurich and Ikeda, respectively).

### Abundance assessment of cryptophytes from metagenomic data

**Fig. 4:**
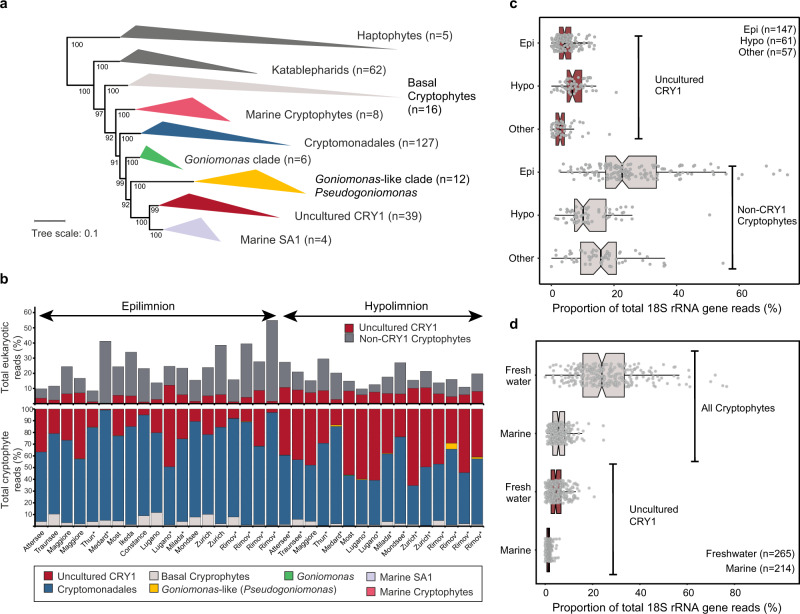
Phylogenetic relationships and abundances of cryptophyte lineages across diverse habitats. **a** Maximum likelihood tree of 18S rRNA gene sequences of cryptophytes. Non-cryptophyte groups (Haptophytes and Katablepharids) are shown in dark gray, Haptophytes were used as outgroup taxa. Ultra-fast bootstrap values are shown at nodes. **b** 18S rRNA abundances of Cryptophyte groups obtained from metagenomic datasets from lakes also used for CARD-FISH analysis. The datasets are ordered based upon Chl-*a* concentrations as shown in Fig. 2. An asterisk (*) indicates metagenomes that do not have corresponding CARD-FISH results. **c** Distribution of % of 18S rRNA reads from uncultured CRY1 and Non-CRY1 cryptophytes in different water layers from metagenomic datasets. The number of datasets used is indicated at top right. The notches in the boxplots indicate confidence intervals around the median. **d** Distribution of % of 18S rRNA gene reads from all cryptophytes and uncultured CRY1 in freshwater and marine metagenomic datasets. The number of datasets used is indicated at bottom right, more details can be found in Supplementary Tables S5–S7.

To assess abundances of cryptophytes in metagenomic data, we elaborated a working classification of known cryptophyte 18S rRNA gene sequences. The phylogenetic affiliation of newly recovered 18S rRNA gene sequences from 479 metagenomic assemblies from freshwater and marine habitats (Supplementary Table S5) led us to establish seven distinct clades in a phylogenetic tree (Fig. 4a): basal cryptophytes (only from freshwaters), marine cryptophytes (only marine), Cryptomonadales (classical photosynthetic cryptophytes), the *Goniomonas* clade (named after the only genome-sequenced species *Goniomonas avonlea*), the “*Goniomonas*-like” clade (or *Pseudogoniomonas* clade, see below), uncultured CRY1 (only freshwater) and Marine SA1 (only marine). Two of these clades included the genus *Goniomonas*, one with three species (*G. avonlea, G. pacifica* and *G. amphinema*) and the other one (“*Goniomonas*-like” in Fig. 4) was represented by *G. truncata*. We also cultured a “*Goniomonas*-like” aplastidic cryptophyte that also gave positive signals with the CARD-FISH probe CryptoB (Fig. 1m, n) and no hybridization was observed with probe Cry1-652 (Fig. 1o, p). Based on its morphological similarity, the phylogenetic proximity of the 18S rRNA gene sequence, and to distinguish it from the *Goniomonas* clade we designate this aplastidic cryptophyte as *Pseudogoniomonas*.

For quantifying abundances of 18S rRNA reads originating from these clades, we compared metagenomic reads from freshwater and marine datasets to a custom SILVA database to which the newly recovered cryptophyte 18S rRNA sequences from metagenomic assemblies were added. Three of the defined groups accounted for most freshwater sequences, i.e., Cryptomonadales (20.1 ± 13.6% of all eukaryotic reads), uncultured CRY1 (5.1 ± 3.6%) and basal cryptophytes (0.7 ± 0.8%, Fig. 4b and Supplementary Tables S6 and S7). Only a small proportion of sequences could be ascribed to the “*Goniomonas*-like” clade (*Pseudogoniomonas*, max. 6.4%). Generally, more cryptophyte sequences were recovered from epilimnetic layers than hypolimnetic ones with maxima of up to 76.7% (average 30.4%, Fig. 4c). On average, (epilimnetic and hypolimnetic wherever available) 27.6% of reads could be assigned to cryptophytes. Conversely, uncultured CRY1 sequences were found in both epilimnion and hypolimnion layers with slightly higher values in hypolimnion datasets (average 7.3%, Fig. 4c). These results also reiterate the relevance of cryptophytes (total average 26%) across diverse freshwater habitats (Fig. 4d and Supplementary Fig. S2). In comparison, marine samples presented much lower read abundances (5.6%). We also found a low proportion of hits to the uncultured CRY1 lineage in some marine open ocean samples although no assembled 18S rRNA sequence that could be ascribed to this lineage was recovered from any marine sample. However, the marine SA1 lineage is the closest phylogenetic neighbor to uncultured CRY1 and may account for these hits. Moreover, while we recovered several 18S rRNA gene sequences of the CRY1 lineage from freshwater metagenomes, none were obtained for the marine SA1 lineage from any marine metagenome. This also suggests that the marine SA1 clade is not abundant in the marine habitat.

## Discussion

### Aplastidic cryptophytes are ubiquitous bacterivores

Our study provides compelling evidence that so far cryptic and largely neglected groups of aplastidic cryptophytes [18, 34, 57] are amongst the most abundant and ubiquitous freshwater bacterivorous HNF (Figs. 1–4). Aplastidic cryptophytes in total accounted for ~2/3 and the CRY1 lineage for 1/4 of all HNF in the epi- and hypolimnion of 24 diverse freshwater habitats (Fig. 2c, f and Table 1). This work extends the conclusions of a previous study conducted only on hypertrophic shallow lakes, suggesting the importance of aplastic bacterivorous cryptophytes as core bacterivores in freshwater habitats [22]. Results based on CARD-FISH counts (Fig. 2) are in line with environmental 18S rRNA gene sequence analyses (Fig. 4), although proportions are lower with the latter method due to uneven rRNA copy numbers in eukaryotes [30]. Our results also differ from classical morphology-based studies attributing the role of major bacterioplankton consumers to other flagellate taxa such as chrysophytes, bodonids and cercozoans [11, 12, 58, 59]. This reflects an overall lack of integration between microscopic and molecular methods [31, 60, 61]. Notably, from aplastidic cryptophytes only the genus *Goniomonas* is usually detectable in DAPI-stained preparations without using FISH-probes. An intriguing question is left to be answered: why did these cryptic aplastidic cryptophyte taxa escape scientist’s attention for such a long time, as 18S rRNA gene sequences affiliated to Cryptophyceae have been recovered in numerous recent studies (e.g., [25, 29, 60, 62]). One reason could be that most aplastidic cryptophytes are of a very small cell size (e.g., CRY1-lineage in Fig. 1a–d) and possess two flagella and thus are barely distinguishable in microscopy-based approaches from typical small *Spumella*-like chrysophytes that have been considered as one of the major freshwater planktonic bacterivores [12, 15, 58]. Our study also shows that aplastidic cryptophytes are generally more abundant (by a factor of 2.5–41; Supplementary Table S2), than their chloroplast-bearing counterparts (e.g., *Rhodomonas*, *Cryptomonas* spp.). However, as chloroplast-bearing cryptophytes are significantly larger than aplastidic ones (Fig. 1q), their biovolume might be higher during seasonally occurring algal blooms. Moreover, chloroplast-bearing cryptophytes were absent in deep hypolimnetic samples, as opposed to abundant aplastidic ones (Fig. 2e), and 18S rRNA gene sequence data clearly shows a presence of Cryptophyceae-related sequences. The most likely explanation of these obvious discrepancies is that compared to other protistan groups (see e.g., [10]) we have insufficient background information on aplastidic cryptophytes (direct observation of cultured species, etc.) and 18S rRNA gene sequence data alone do not provide information on trophic modes or the presence or absence of chloroplasts in cells [22, 31]. Thus, microscopic determination of chloroplasts and/or of ingested prey types in food vacuoles of FISH-stained flagellates might become an important part of future protistan research.

We also used FLB in combination with CARD-FISH in seven lakes (Table 1), showing significantly higher uptake rates of larger CryptoB-targeted flagellate cells, compared to small CRY1 cells (Fig. 3, see also [22]). While bacterivory of chloroplast-bearing cryptophytes has been frequently reported in previous studies of freshwater [26, 63, 64] and marine systems (with an occurrence of smaller forms of plastidic cryptophytes [32]), we did not observe any uptake of tracer FLB by CryptoB-positive, large chloroplast-bearing cryptophyte cells in our samples.

Thus, their grazing effect was likely negligible compared to the far more abundant aplastidic cryptophytes ingesting bacteria, which in line with our study hypothesis—represented voracious bacterivores in all studied lakes and depths (Figs. 1–3).

Our results clearly show that aplastidic cryptophytes and CRY1 are ubiquitous bacterivores and, moreover, their abundances correlated with abundances of their prokaryotic prey in the epilimnion of the studied habitats (Supplementary Table S3). Chl-*a* concentrations, used as a proxy of system trophic states, tightly correlated with abundances of bacterivorous aplastidic cryptophytes and of the CRY1 lineage in epilimnetic water layers. In contrast, both abundances and relative proportions of these cryptophyte groups were not correlated with bacterial abundances in hypolimnetic samples (Supplementary Table S4), although ingested bacteria were always observed in the protists’ food vacuoles. This trophic coupling seems to be more complex in the generally colder hypolimnetic waters with less abundant but larger bacteria and more suspended lake snow particles [65]. Since the general probe CryptoB targets all Cryptophyceae, we can only speculate about their different adaptation to selective feeding on larger or particle-associated bacteria in deeper strata of lakes, a feeding strategy known for e.g., kinetoplastids. Kinetoplastids have been shown to be abundant in deep oxygenated lake waters during periods of high concentrations of lake snow particles [66, 67], where they can feed on surface-associated bacteria [68, 69]. We included four shallow hypertrophic lakes (Table 1) in our data set, that, besides the high turbidity and extreme Chl-*a* concentrations (Fig. 2a), contain a lot of suspended particles overgrown by bacteria, thus forming a trophic niche for large populations of kinetoplastids [22]. On the other hand, relative proportions of CryptoB (Fig. 2c) were slightly lower in these hypertrophic systems compared to most oligo-mesotrophic and meso-eutrophic lakes. This might hint at a different feeding strategy of aplastidic cryptophytes in comparison to kinetoplastids.

While relative proportions of aplastidic CryptoB were relatively stable in the lakes analyzed here, the CRY1 lineage showed a large variability in absolute and relative proportions (Fig. 2). These HNF are of small cell sizes (Fig. 1a–d and Supplementary Table S2), having a high growth potential with doubling times of hours to days [22, 34]. This might indicate their rapid population turnover time in plankton environments, where they can efficiently control fast-growing prokaryotic lineages. We hypothesize that their generally higher proportions in lakes of lower trophic states (oligo- to meso-eutrophy, Fig. 2c), poor in bacterial aggregates, might predefine them as prominent consumers of rapidly growing suspended bacterial taxa forming short-lived abundance peaks associated with algal blooms [40, 70].

### A need for more cultivated representatives of aplastidic cryptophytes

The critical issue of testing ecological hypotheses is hampered by an almost complete lack of isolated representatives of aplastidic cryptophytes. Traditional protist cultures have been mainly obtained using nutrient-rich media (e.g., grain infusions) that might mimic naturally occurring nutrient-rich hotspots [71]. Such approaches, while effective, have also introduced a recovery bias toward certain lineages and new cultivation approaches need to be developed. Among known aplastidic lineages, the uncultured CRY1 lineage accounts for a large fraction, while others (e.g., *Goniomonas* and *Pseudogoniomonas*) are far less prevalent (Fig. 4 and Supplementary Fig. S2) and additional unknown taxa of aplastidic cryptophytes seem to be ubiquitous and highly abundant (Fig. 2). However, apart from photosynthetic Cryptomonadales (*Cryptomonas*, *Rhodomonas, Teleaulax, Geminigera, Guillardia, Hanusia, Proteomonas*) and non-photosynthetic *Goniomonas, Chilomonas, Hemiarma (marine SA1 clade)* and the here newly cultured *Pseudogoniomonas*, no other lineages have any cultivated representatives.

A potential confounding factor may be the misidentification of cryptophytes by isolators that leads to anomalies in 18S rRNA gene sequence databases. For example, *Teleaulax amphioxeia* 18S rRNA gene sequences (AB364287) can be found within the classical Cryptomonadales lineage, but also appear in the Basal Cryptophyte lineage (Fig. 4). If the organism classified as *Teleaulax* in the Basal Cryptophyte lineage is photosynthetic, it might imply that all members of this lineage are also likely photosynthetic. Unfortunately, no information could be found for this isolate. Another confounding factor is the recent discovery that two different photosynthetic cryptophyte genera, *Teleaulax* and *Plagioselmis* represent a case of morphological dimorphism, and are actually the same organism [72], *Plagioselmis* being the haploid life stage of the diploid *Teleaulax*. Similar dimorphism has been reported for *Geminigera* [73] as well.

Members of the genus *Goniomonas* [23] might be another example of such potential anomaly. For instance, we cultured what appeared to be aplastidic *Goniomonas* by morphology (Fig. 1m–p). However, 18S rRNA gene sequencing of this culture revealed that it belonged to the *Goniomonas*-like clade. Similar isolates have been obtained before and nominated as *Goniomonas* (sp. *truncata*). However, even though *Pseudogoniomonas-*like sequences appeared to be more prevalent than those of *Goniomonas*, they were still insufficient to account for the abundant and morphologically diverse morphotypes of aplastidic non-CRY1 cryptophytes (Fig. 1e–h, compare also [22]) that are repeatedly observed at multiple sites and remain an unsolved mystery.

### Diverse unknown aplastidic cryptophytes might still await discovery

As of now only the Cryptomonadales lineage is known to include photosynthetic cryptophytes (Fig. 4). This lineage also contains the non-photosynthetic species *Chilomonas paramecium*, a relatively large (ca 15–20 µm long) flagellate known from benthic samples [11] and phytothelmata of bromeliad [74], with the ability to grow on bacterial and detrital particles [24]. The other known non-photosynthetic cryptophyte clades are uncultured CRY1, *Goniomonas*, and *Pseudogoniomonas*. No reliable information is available for other clades that are known only from sequence data or from cells being visualized by the general probe CryptoB (Fig. 1e–h). Interestingly, it has been speculated based upon genomic data that *Goniomonas* never acquired photosynthetic capacity [75], while non-photosynthetic *Chilomonas* have reduced their chloroplasts to leucoplasts [76]. Which scenario turns out to be true for uncultured CRY1 will become evident only once a culture and/or genome become available. Given the diverse and relatively larger cell sizes of non-CRY1 aplastidic cryptophytes (Fig. 1) and also their high abundances as observed by CARD-FISH (Fig. 2), it may be that the Cryptomonadales lineage (that is also the most abundant, Fig. 4) involves additional uncultured “cryptic” aplastidic cryptophytes that are indistinguishable using 18S rRNA gene sequences.

## Conclusions

This study exemplifies the powerful combination of CARD-FISH and environmental sequence analyses, which show that aplastidic bacterivorous Cryptophyceae are widely distributed across lakes of different trophic states, pinpointing them as major freshwater bacterivorous flagellates linking carbon flows from prokaryotes to higher trophic levels. So far, Cryptophyceae have been considered to contain predominantly chloroplast-bearing representatives with only moderate capabilities to switch from autotrophy to phagotrophy of bacteria, and only a few heterotrophic lineages are known. Our study shows a worldwide ubiquity and high abundance of heterotrophic cryptophytes, which, except for the well-defined CRY1 lineage, might contain a large diversity of so far cryptic bacterivorous species of very diverse morphologies and cell sizes that still await a better taxonomic and ecological characterization.

## Supplementary information

Supplementary Figures S1 and S2

All_Supplementary_Tables - Simek.xlsx

## Data Availability

All sequences used in the tree, their alignment and the final phylogenetic tree are available for download in Figshare (https://figshare.com/s/8540087c6bf5464ff17f).
