# Supplementary material for: Cryptic and ubiquitous aplastidic cryptophytes are key freshwater flagellated bacterivores
Source: ISME J. 2022 Oct 7;17(1):84–94. doi: 10.1038/s41396-022-01326-4 (PMC9751141; doi:10.1038/s41396-022-01326-4)
Supplement: Supplementary file 1 — Supplementary Figures S1 and S2 [file 41396_2022_1326_MOESM1_ESM.pdf]

Supplementary Information

Cryptic and ubiquitous aplastidic cryptophytes are key freshwater flagellated bacterivores

Karel Šimek, Indranil Mukherjee, Tiberiu Szöke-Nagy, Markus Haber, Michaela M. Salcher, Rohit Ghai

----

Supplementary Figure S1. Lake Biwa: (**a**) One year seasonal course of Chl-*a* concentrations and total abundance of bacteria and HNF, of absolute (**b**) and relative proportions (**c**) of aplastidic cryptophytes targeted by probe CryptoB and by probe Cry1-652 (targeting its Cry1 lineage) in total HNF.

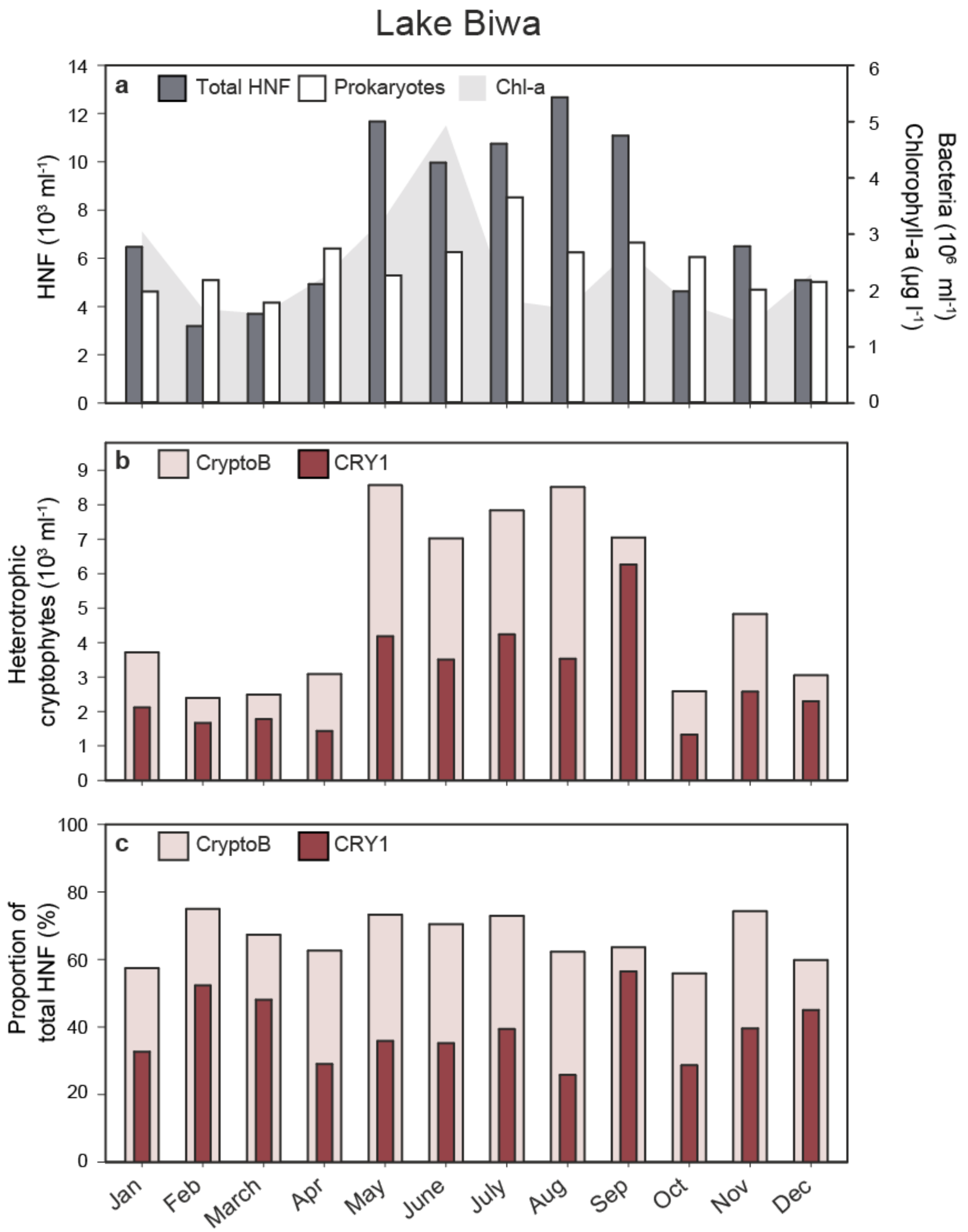

Supplementary Figure S2: (a-f) 18S rRNA abundances (expressed as percentage of total eukaryotic reads) of CRY1 and non-CRY1 cryptophytes across metagenomes from diverse freshwater habitats. The site names are indicated at top left for each. Wherever known the sampling depths are indicated. Legend is at bottom right.

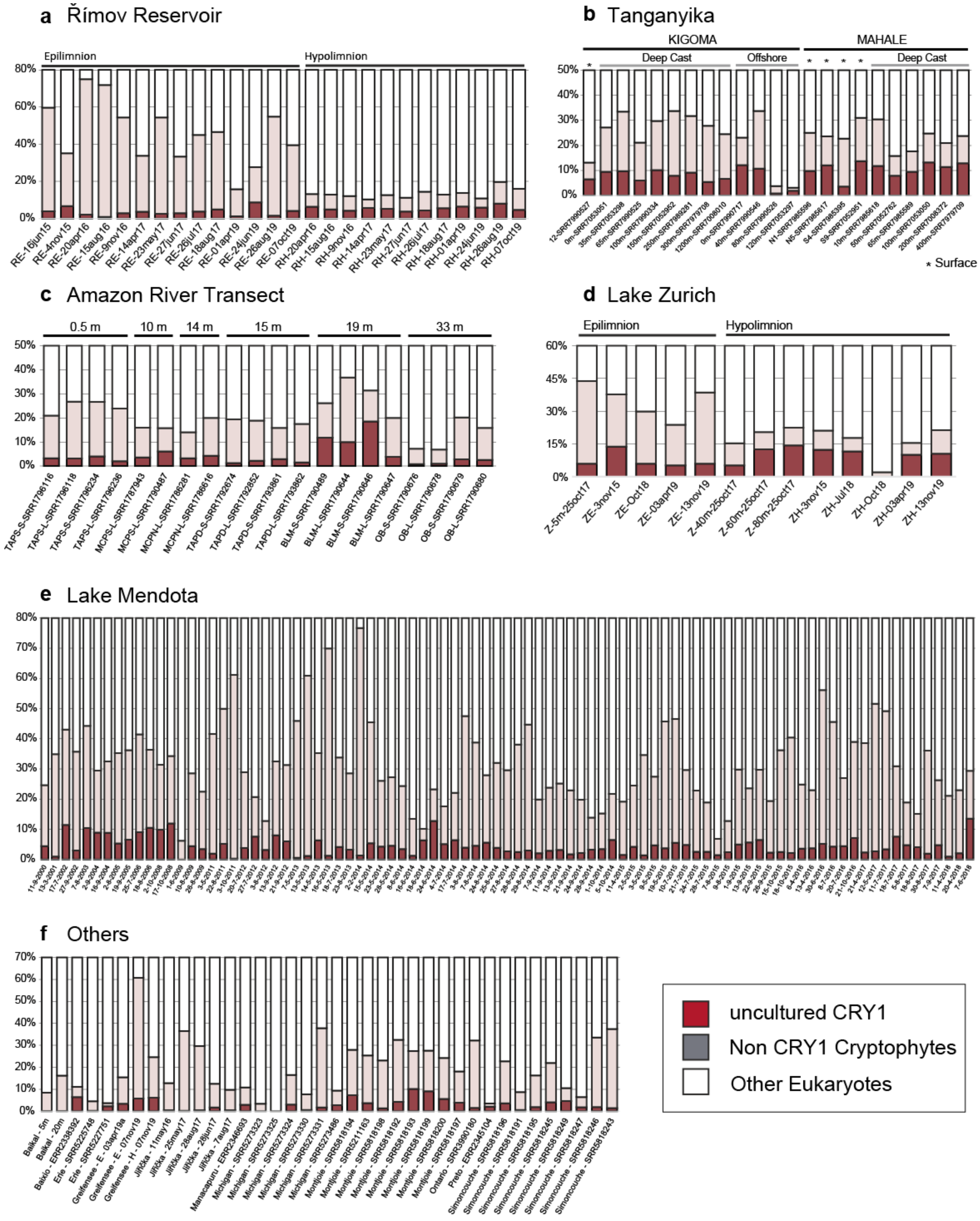

Supplementary Tables can be found as separate file (All_Supplementary_Tables - Simek.xlsx)

Supplementary Table S1. Characteristics (country of origin, date and depth of sampling) and a total number of samples analyzed from the epilimnion of 24 lakes and the metalimnion of 14 deep lakes. Hypolimnetic samples were mostly taken ca. 10 m above the bottom of the lake.

Supplementary Table S2. Abundances, mean cell volumes and numerical and biovolume ratios of aplastidic to plastidic cryptophytes from the epilimnion of four oligo-mesotrophic lakes with a simultaneous occurrence of both aplastidic (see examples in Fig. **1a**–**h**) and chloroplast-bearing (*Rhodomonas* and *Cryptomonas* spp.; Fig. **1i**–**l**) cryptophytes. Cryptophytes were targeted by probes CryptoB and Cry1-652. The percentage of CRY1 to total aplastidic cryptophytes is given in parentheses.

Supplementary Table S3. Spearman's rank correlation coefficients of determination (R^2) of the data from the epilimnion of 24 lakes shown in Fig. **2a–c**. % - relative proportions of probe-defined groups of cryptophytes; abund. – abundances of bacteria, total HNF and flagellates targeted by probes Cry1-652 and CryptoB. Significant positive correlation are in black bold, significant negative correlation in red bold; * $p < 0.05$, ** $p < 0.01$, *** $p < 0.001$.

Supplementary Table S4. Spearman's rank correlation coefficients of determination (R^2) of the data from the hypolimnion of 14 lakes shown in Fig. **2d–f**. % - relative proportions of probe-defined groups of cryptophytes.; abund. – abundances of bacteria, total HNF and flagellates targeted by probes Cry1-652 and CryptoB. Significant positive correlation are in bold; * $p < 0.05$, ** $p < 0.01$, *** $p < 0.001$.

Supplementary Table S5. Metagenomic datasets and sampling details for the analyzed metagenomes. "N / A" stands for not available.

Supplementary Table S6. Number of 18S rRNA reads recovered from freshwater metagenomic datasets. 20 million reads were sampled from each dataset, only those that had >500 eukaryotic 18s rRNA hits are shown here. (*) Entire dataset was scanned for 18s rRNA sequences to identify Cryptophytes.

Supplementary Table S7. Number of 18S rRNA reads recovered from metagenomic datasets which also have CARD-FISH data.
